# Evaluation of Electrical Cardiometry for Measuring Cardiac Output and Derived Hemodynamic Variables in Comparison with Lithium Dilution in Anesthetized Dogs

**DOI:** 10.3390/ani13142362

**Published:** 2023-07-20

**Authors:** Vaidehi V. Paranjape, Fernando L. Garcia-Pereira, Giulio Menciotti, Siddharth Saksena, Natalia Henao-Guerrero, Carolina H. Ricco-Pereira

**Affiliations:** 1 Department of Small Animal Clinical Sciences, Virginia-Maryland College of Veterinary Medicine, Blacksburg, VA 24061, USA; giuliom@vt.edu (G.M.); nguerrer@vt.edu (N.H.-G.); 2 Pet Urgent Response and Emergency, Jacksonville, FL 32256, USA; veterinaryanesthesiaservices@gmail.com; 3 Department of Civil and Environmental Engineering, Virginia Polytechnic Institute and State University, Blacksburg, VA 24061, USA; ssaksena@vt.edu; 4 Department of Veterinary Clinical Sciences, The Ohio State University-College of Veterinary Medicine, Columbus, OH 43210, USA; riccopereira.1@osu.edu

**Keywords:** canine, isoflurane, dobutamine, esmolol, phenylephrine, noninvasive, electrical velocimetry, pulmonary artery thermodilution, inotrope, vasopressor

## Abstract

**Simple Summary:**

Cardiac output (CO) measurement devices are classified as invasive, minimally invasive, or noninvasive depending on their level of invasiveness for CO data acquisition. Pulmonary artery thermodilution is the ‘gold standard’ CO technique. This method is more accurate, but its invasiveness possesses risks. Minimally invasive lithium dilution (LiD) is slowly replacing thermodilution as a reference standard in animal research due to its excellent agreement and acceptable performance. Monitoring CO with standard cardiovascular parameters (i.e., blood pressure and heart rate) in anesthetized animals can potentially improve patient care and case outcomes. Hence, we evaluated noninvasive electrical cardiometry (EC)-measured CO and other EC-acquired hemodynamic variables, and analyzed them against CO measured using LiD in healthy, anesthetized dogs during different treatments (dobutamine, esmolol, phenylephrine, and high-dose isoflurane) impacting CO values. Overall, EC showed good agreement with LiD, but it exhibited consistent underestimation when the CO values were higher. The percentage error was low and within published standards, and a good trending pattern was exhibited by EC. The acquired EC variables followed the trends in CO obtained by LiD. EC may be a pivotal tool for monitoring trends in hemodynamics and guiding treatments for cardiovascular anesthetic complications in clinical settings.

**Abstract:**

Numerous cardiac output (CO) technologies were developed to replace the ‘gold standard’ pulmonary artery thermodilution due to its invasiveness and the risks associated with it. Minimally invasive lithium dilution (LiD) shows excellent agreement with thermodilution and can be used as a reference standard in animals. This study evaluated CO via noninvasive electrical cardiometry (EC) and acquired hemodynamic variables against CO measured using LiD in six healthy, anesthetized dogs administered different treatments (dobutamine, esmolol, phenylephrine, and high-dose isoflurane) impacting CO values. These treatments were chosen to cause drastic variations in CO, so that fair comparisons between EC and LiD across a wide range of CO values (low, intermediate, and high) could be made. Statistical analysis included linear regression, Bland–Altman plots, Lin’s concordance correlation coefficient (ρ_c_), and polar plots. Values of *p* < 0.05 represented significance. Good agreement was observed between EC and LiD, but consistent underestimation was noted when the CO values were high. The good trending ability, ρ_c_ of 0.88, and low percentage error of ±31% signified EC’s favorable performance. Other EC-acquired variables successfully tracked changes in CO measured using LiD. EC may be a pivotal hemodynamic tool for continuously monitoring circulatory changes, as well as guiding and treating cardiovascular anesthetic complications in clinical settings.

## 1. Introduction

Cardiovascular complications frequently emerge during general anesthesia. Intraoperative patient management can be challenging due to these acute hemodynamic alterations [1]. Routinely, inhalant anesthesia is incorporated into the anesthetic protocol for maintenance of general anesthesia. Acute cardiovascular effects from inhalant anesthetics can be focused on: (i) myocardial function, (ii) electrophysiologic pattern, (iii) regulation of coronary blood flow, and (iv) systemic and pulmonary vasoregulation [2].

It is largely known that inhalants induce myocardial depression in a dose-dependent fashion, mainly by regulating sarcolemmal L-type Ca^++^ channels, the sarcoplasmic reticulum, and contractile proteins [2,3]. Experimental canine studies reveal that different inhalant anesthetics do not have the same impact on the myocardium, and that halothane and enflurane have more potent negative chronotropic effects than isoflurane, desflurane, or sevoflurane [4,5]. Studies in dogs indicate that the order for myocardial sensitization to arrythmias is halothane > enflurane > sevoflurane = isoflurane [6,7,8,9]. All halogenated agents decrease systemic blood pressure in a dose-dependent manner due to a reduction in either stroke volume and CO or systemic vascular resistance, or a combination of these factors [10,11,12]. Clinicians have reported the association between intraoperative hypotension and its duration with postoperative mortality and organ dysfunction after general anesthesia [13]. Endothelial-dependent mechanisms include the effect on bradykinin levels, ATP- and histamine-induced Ca^++^ influx, activation of endothelial nitric oxide synthase, release of nitric oxide, endothelin-1 production, angiotensin II-induced vascular smooth muscle contraction, Ca^++^ sensitivity, and the calcium-induced calcium release process [2]. Additionally, each element of the baroreceptor reflex arc can be inhibited by inhalants [14]. Agent-specific alterations in the basic pulmonary vascular tone and vasoactive regulation of the pulmonary vasculature were also addressed [15,16].

Cardiovascular monitoring and management are key pillars of perioperative patient care. The end goal is always focused on the optimization of oxygen delivery to the tissues. Along with variables such as blood pressure and heart rate, there is a need for precise quantification of cardiac output (CO) in operative rooms, trauma units, and intensive care units which is critical for generating therapeutic protocols that can enhance oxygen delivery. Innovative minimally invasive and noninvasive CO monitoring tools require prior validation before they can be incorporated into clinical settings to improve perioperative care. A reduction in morbidity and mortality can be achieved by combining advanced hemodynamic monitoring with guided therapeutic decisions and continuous assessments in high-risk patients [17]. In veterinary species, minimally invasive CO methods, such as lithium dilution (LiD), transpulmonary thermodilution, pulse contour analysis, pulse pressure analysis, and many others were studied [18,19]. Even though the accepted gold standard for CO measurement in veterinary medicine is still pulmonary artery thermodilution, clinicians are hesitant to use this technique in research and clinical settings due to the associated risks and invasiveness, required training and skill, and costs for instrumentation. Using various animal models, LiD was shown to be a precise and acceptable alternative to the gold standard technique [20,21,22,23,24,25].

Electrical cardiometry (EC) is a relatively newer noninvasive technology that can continuously assess CO and other hemodynamic parameters such as heart rate (EC_HR_), stroke volume (EC_SV_), systemic vascular resistance (EC_SVR_), thoracic fluid content, corrected flow time (FTC), stroke volume variation, contractility index (ICON™), variation in contractility, systolic time ratio (STR), pre-ejection period (PEP), and left ventricular ejection time (LVET). The successful tracking of CO using EC and an overall acceptable performance were highlighted in numerous studies in critically ill adults and children [26,27,28]. However, there are limited data in veterinary medicine signifying the ability of EC to measure CO and trace cardiovascular changes via EC-derived parameters in anesthetized dogs [29,30,31,32,33] and pigs [34]. The physiologic model installed in the ICON monitor is called Electrical Velocimetry™ (Osypka Medical Inc., La Jolla, CA, USA), which detects variations in thoracic electrical bioimpedance during aortic ejection and analyzes the volumetric changes in the aorta and orientation of the erythrocytes varying with the cardiac cycle [35,36]. Electrical alternating current of a constant amplitude is released in the direction of the aorta due to blood being a significant conductive material in the thoracic cavity. The ratio of applied current and measured voltage is equivalent to the thoracic electric bioimpedance. The physiological theory behind EC is that during a cardiac cycle, the alignment of erythrocytes residing in the aorta changes, which further induces differences in the impedance. During cardiac diastole, before the aortic valve opens, the erythrocytes display random orientation within the aorta due to lack of flow, which coincides with a higher voltage and impedance recording. During cardiac systole, after the aortic valve opens, the flow converts to being pulsatile, which corresponds to a lower impedance detected by the monitor. Using the variability in thoracic electrical bioimpedance, EC derives multiple hemodynamic parameters. The detailed algorithm and mechanism are well discussed in the literature [33,34,35,36].

The effect of pharmacological interventions on cardiac contractility and afterload further impacting EC-acquired CO and other variables is not yet studied. During pharmacological maneuvering of hemodynamics in anesthetized, healthy dogs, the specific aims of our study were to: (i) evaluate the level of agreement in CO measurement between LiD and EC, and (ii) quantify the relationship between EC-acquired variables, i.e., EC_SV_, EC_SVR_, FTC, ICON™, STR, PEP, and LVET with respect to CO measured using LiD and invasive arterial blood pressure. We hypothesized that: (i) EC will share an acceptable level of agreement with LiD, and (ii) EC-acquired variables will be able to track the sudden, treatment-induced changes in the CO and MAP values.

## 2. Materials and Methods

### 2.1. Animals

This prospective experimental design utilized six adult purpose-bred, male, healthy beagles (aged 1–2 years; weighing 11.5 ± 0.9 kg). This was a crossover, randomized research study. A thorough physical examination, complete blood count, and serum chemistry panel were performed to categorize the dogs under American Society of Anesthesiologists Physical status 1. The experimental study procedures and animals used were in concordance with the Virginia Tech University Institutional Animal Care and Use Committee (protocol number 20-229). A month after the end of the study, all dogs were adopted into single-family homes.

An a priori power analysis was conducted considering the prior animal studies, which evaluated test methods for CO and compared them to a reference CO method [33,37,38,39,40], and it was concurred that six animals would be required to detect a 30% significant difference in CO in response to hemodynamic manipulation, setting a statistical power of 0.8 and an alpha level of 0.05 (http://estatistica.bauru.usp.br/calculoamostral/; accessed on 24 January 2021).

### 2.2. Induction of General Anesthesia and Standard Anesthetic Monitoring

All dogs were allowed to familiarize themselves with the laboratory space and were acclimatized for two weeks prior to starting the research. The dogs were fasted for solid food for 12 h but had water access. On the day of the experiment, aseptic cephalic catheter placement was carried out, followed by oxygen supplementation with a facemask attached to an anesthesia machine using a circle breathing system (4 L/min) for five minutes. Intravenous propofol was administered in titration until orotracheal intubation was achieved. The cuffed endotracheal tube was secured and connected to the circle breathing system and a ventilator-integrated anesthesia workstation (Datex-Ohmeda Aestiva 5/7900; GE Healthcare, Chicago, IL, USA). The dogs were then transitioned to the dorsal recumbency. Isoflurane in oxygen (1.5–2.5 L/min) was selected for anesthetic maintenance. The end-tidal concentration of isoflurane (ET_ISO_) was continuously monitored with an infrared gas analyzer included in a multiparameter monitor and was targeted at 1.4–1.6%. Standard anesthetic monitoring comprised a lead II electrocardiogram for heart rate and rhythm, pulse oximeter, capnography, and esophageal temperature recorded using the same monitor. Normothermia (36.7 to 38 °C) was maintained in all dogs throughout the anesthetic period. These variables were recorded every five minutes as part of the standard anesthesia monitoring throughout the anesthetic period. No maintenance fluids were infused to prevent their influence on blood volume, which could impact the hemodynamic data.

Volume-controlled ventilation using a constant tidal volume of 12 mL/kg and respiratory rate adjusted to maintain the end-tidal carbon dioxide concentration between 30 and 40 mmHg were set as ventilation settings. Aseptic arterial catheterization in the dorsal pedal artery of both hindlimbs was performed, with one arterial line dedicated to CO measurements using LiD, and the other line for measuring invasive systolic, diastolic, and mean arterial blood pressure (MAP). This arterial catheter was attached to a disposable pressure transducer system that was pre-flushed with heparinized saline (3 IU/mL) and was leveled and zeroed at the level of the heart. An IV catheter was placed aseptically in the jugular vein on the left side, which was used for treatment administration and LiD measurements of CO. A 6 Fr 8.5 cm hemostasis introducer was aseptically inserted by performing a modified Seldinger technique in the right jugular vein, through which a 5 Fr 75 cm Swan Ganz thermodilution catheter was advanced into the PA by observing pressure waveforms. The hemodynamic data from the thermodilution technique were collected for another clinician’s research project, but the catheter was used to measure right atrial pressure to derive EC_SVR_ data for the present study. All dogs were positioned in the right lateral recumbency for the EC setup. Due to this postural change, the arterial pressure transducer was readjusted to align with the level of the right atrium and re-zeroed.

### 2.3. Instrumentation for EC to Measure CO (CO_EC_) and Other Hemodynamic Variables

**Figure 1:**
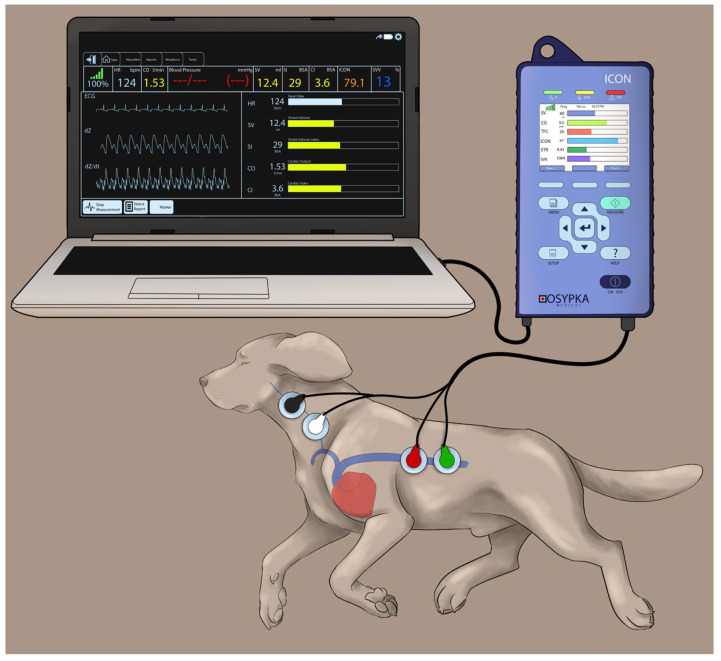
The Cardiotronic electrodes are placed on a study dog positioned on the right lateral recumbency and connected to an electrical cardiometry monitor (ICON; Osypka Medical Inc., La Jolla, CA, USA). Out of the four electrodes, two of them are located on the left side of the neck adjacent to the common carotid artery, and the other two are placed on the left lower chest wall. The electrodes are attached to the ICON monitor via a cable, and the monitor is synced to a laptop for data display and storage. The image was reproduced with permission from Paranjape V.V., journal *Animals*, published by MDPI (2023) [33].

The electrical cardiometry setup consisted of an ICON monitor (Osypka Medical Inc., La Jolla, CA, USA) connected to four Cardiotronic (Osypka Medical Inc.) electrocardiographic electrodes and a separate cable connected to a laptop for syncing the data via the iControl™ application (Osypka Medical Inc.) for data display and storage (Figure 1). After clipping a 4 × 4 cm area on the left side of the neck alongside the common carotid artery and a 5 × 5 cm area on the left lower aspect of the thorax, both areas were wiped clean. Once these areas were dry, using the adhesive patch, two electrodes were located on the neck at the level of the common carotid artery, while two other electrodes were placed on the left chest wall paralleling T8–T13 vertebrae and coinciding with the descending thoracic aorta location [33].

By assessing the rate of change in impedance during one cardiac cycle, the ICON algorithm estimates stroke volume (SV_EC_), which is then multiplied by heart rate (HR_EC_) to yield the CO values (EC_CO_). To ensure the reliability of the EC data, the HR_EC_ values were verified against the pulse rate from the pulse oximetry and arterial pressure waveforms as well as the HR from the electrocardiogram before recording CO_EC_, SV_EC_, HR_EC_, and other EC-acquired hemodynamic variables. We ensured that EC data were only collected when the signal quality index displayed on the ICON monitor was 100 to assure accuracy. The EC_SVR_ data were calculated using the ICON monitor by feeding the MAP values along with the right atrial pressure measurements from the thermodilution catheter with the following equation [37]. HR_EC_, SV_EC_, CO_EC_, and EC_SVR_ were averaged over a one-minute interval as set in the internal database.

$${\mathrm{EC}}_{\mathrm{SVR}}\;(\mathrm{dyn}•s/{\mathrm{cm}}^{5})=\;\frac{(\mathrm{Mean}\;\mathrm{Arterial}\;\mathrm{Pressure}\;-\mathrm{Right}\;\mathrm{Atrial}\;\mathrm{Pressure})\;\times \;80}{\mathrm{Cardiac}\;\mathrm{output}\;\mathrm{by}\;\mathrm{Electrical}\;\mathrm{Cardiometry}}$$

### 2.4. Instrumentation for LiD to Measure CO (LiD_CO_)

**Figure 2:**
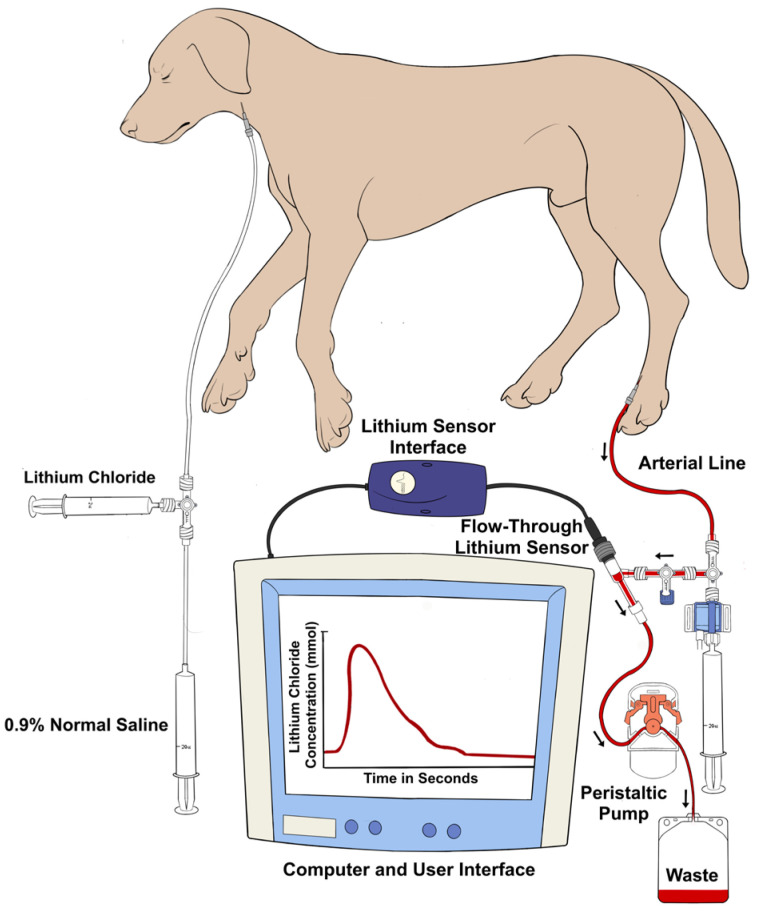
Graphical illustration of the venous and arterial instrumentation for the lithium dilution technique. Isotonic lithium chloride is injected into a central vein, and a concentration–time curve is generated by an ion-selective electrode attached to the dorsal pedal arterial line pressure transducer system. The area under the curve of the plot of the lithium concentration against time allows for the calculation of the cardiac output.

A LiDCOplus monitor (LiDCO Ltd., Cambridge, UK) was used to perform the LiD_CO_ measurements. The setup consisted of arterial and venous instrumentation (Figure 2) [21]. On the arterial side, once the lithium-sensitive sensor was primed and flushed with 0.9% normal saline, its inlet port was attached to one of the ports of a three-way stopcock connected to the arterial catheter via a disposable pressure transducer system and noncompliant tubing. The other end of the sensor was fused with the lithium sensor interface, which was directly synced with the LiDCOplus monitor via a cable. The outlet port of the sensor was attached to a disposable blood collection waste bag with the help of soft extension tubing. The tubing between the sensor and the collection bag passed through a peristaltic flow regulator pump. Once the pump switch was turned on and the stopcock was opened, the blood flowed from the dorsal pedal artery across the sensor at a constant rate (4 mL/min), eventually leading it to the waste bag. The specific sensor constant was typed into the LiDCOplus computer. On the venous side, the left jugular catheter was connected to extension tubing and a three-way stopcock. A labeled syringe containing 0.004 mmol/kg lithium chloride bolus (LiDCO Ltd., Cambridge, UK) [24,41,42] was attached to one port of the stopcock, while the other port was connected to a 20 mL syringe containing 0.9% normal saline. Once ready for CO measurement, a stable baseline was confirmed on the computer screen. After pressing ‘inject’ on the screen, lithium chloride was injected, followed by the saline flush. The injection was quick, using a constant, firm pressure. A smooth upstroke of the dilution curve was verified on the computer screen, and once approved, the monitor indicated to turn the pump off and flush the system.

The CO calculation by the LiDCOplus monitor, as shown below [20,41,42,43], was based on the lithium dose used and the area under the concentration–time dilution curve with the help of internal software.

$${\mathrm{LiD}}_{\mathrm{CO}}=\;\frac{\mathrm{Lithium}\;\mathrm{chloride}\;\mathrm{dose}\;(\mathrm{mmol})\;\times \;60}{\mathrm{Area}\;\mathrm{of}\;\mathrm{curve}\;\mathrm{corrected}\;\mathrm{for}\;\mathrm{sodium}\;\mathrm{concentration}\;(\mathrm{mmol}/L)\;\times \;(1\;-\;\mathrm{packed}\;\mathrm{cell}\;\mathrm{volume})}$$

Vital information required by the monitor includes the hemoglobin and plasma sodium concentrations, along with the lithium dose. The lithium-sensitive electrode has a selective membrane through which only lithium can penetrate, and the voltage across the sensor membrane is related to the plasma lithium concentration via the Nernst equation. Since in the absence of lithium the baseline voltage is dependent on the plasma sodium concentration, this correction is applied. Serial measurements of blood hemoglobin, packed cell volume, and plasma sodium concentration were determined in each hemodynamic phase by using a benchtop blood gas analyzer (i-STAT with CHEM8+ cartridge; Abbott Point of Care, Princeton, NJ, USA) prior to each LiD estimation. These data were fed into the LIDCO monitor each time the first CO measurement was carried out during every treatment that changed hemodynamic conditions. For every dog, a new LiD_CO_ sensor was used to avoid interactions with the previous setup. The LiD_CO_ measurement used in each analysis corresponded to the mean of two consecutive observations obtained via dilution curves produced with lithium injection, which were within 10% variation of each other.

### 2.5. Administration of Treatments, Associated Hemodynamic Goals, and Data Collection

**Figure 3:**
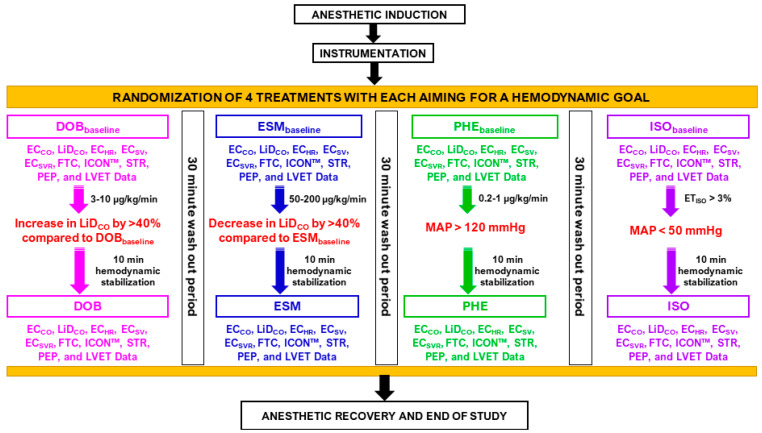
Data collection during the experimental design in six isoflurane-anesthetized beagles receiving four treatments. Cardiac output data via electrical cardiometry (EC_CO_) and lithium dilution (LiD_CO_), and other EC-acquired indices, such as heart rate (EC_HR_), stroke volume (EC_SV_), systemic vascular resistance (EC_SVR_), corrected flow time (FTC), contractility index (ICON™), variation in contractility, systolic time ratio (STR), pre-ejection period (PEP), and left ventricular ejection time (LVET). Baseline values were noted before dobutamine (DOB_baseline_), esmolol (ESM_baseline_), phenylephrine (PHE_baseline_), and high-dose isoflurane (ISO_baseline_) treatments: (i) DOB: dobutamine IV infusion 3–10 μg/kg/min to cause an elevation in LiD_CO_ by >40% versus DOB_baseline_; (ii) ESM: esmolol IV bolus 100 μg/kg followed by infusion 50–200 μg/kg/min to lower LiD_CO_ by >40% versus ESM_baseline_; (iii) PHE: phenylephrine IV infusion 0.2–1 μg/kg/min for mean arterial pressure >120 mmHg; and (iv) ISO: end-tidal isoflurane concentration >3% for mean arterial pressure <50 mmHg. Data were obtained after ten minutes of hemodynamic stabilization and at least post 30 min wash-out period between interventions. Start of intervention and baseline recordings were performed only when there was <10% variation in LiD_CO_ baseline data of two treatments.

All dogs underwent four treatments (Figure 3) in a randomized order (https://www.randomizer.org/; accessed on 10 March 2021). Baseline values were noted before initiating treatments, i.e., before dobutamine (DOB_baseline_), esmolol (ESM_baseline_), phenylephrine (PHE_baseline_), and high-dose isoflurane (ISO_baseline_) treatments. It was ensured that minimal variation existed between the baseline data of the four treatments; hence, the LiD_CO_ measurements were used to guide when to start the interventions. The decision to initiate the next intervention and recording of baseline data was performed only when there was <10% variation between baseline LiD_CO_ data obtained via lithium injection between treatments. Pre-established target values were in place to ensure a significant change in the hemodynamics was observed that was specific to each treatment. These goals for each of the four treatments were as follows. (i) DOB: dobutamine (12.5 mg/mL) IV infusion 3–10 μg/kg/min to increase LiD_CO_ by >40% versus DOB_baseline_; (ii) ESM: esmolol (10 mg/mL) IV bolus 100 μg/kg followed by infusion 50–200 μg/kg/min to decrease LiD_CO_ by >40% versus ESM_baseline_; (iii) PHE: phenylephrine (10 mg/mL) IV infusion 0.2–1 μg/kg/min for MAP > 120 mmHg; and (iv) ISO: ET_ISO_ > 3% for MAP < 50 mmHg.

Ten minutes were reserved for stabilization after reaching the hemodynamic goal corresponding with each intervention and before any data were obtained. A minimum of 30 min were allotted as the wash-out period between treatments to restrict any carryover circulatory effects of the prior drug from skewing the results. Each researcher recorded CO data from either EC or LiD and they were blinded to each other. One researcher was responsible for initiating and stopping treatments. The sequence for obtaining CO data was always EC_CO_ followed by LiD_CO_, to prevent the saline injections and waste arterial volume from LiD affecting the EC_CO_ measurements and EC-derived hemodynamic variables. The CO data from the two techniques and other hemodynamic data were obtained at the end of the expiration.

### 2.6. Recovery from General Anesthesia

Once final data were procured, the jugular and arterial catheters were carefully removed, and external pressure was applied on the catheter sites to prevent bleeding and hematoma formation. Isoflurane vaporizer was turned off to begin anesthetic recovery. A methadone dose of 0.2 mg/kg IV was administered to all dogs before transferring them to individual kennels. Vigilant monitoring of cardiopulmonary parameters and pain assessment using the Glasgow composite pain scale short form was conducted frequently for the next 96 h. Another 0.2 mg/kg IV methadone dose was administered if required based on the pain scores.

### 2.7. Statistical Analysis

The hemodynamic variables in relation to different interventions were assessed for normality using the Shapiro–Wilk and D’Agostino–Pearson tests and data were presented as mean ± standard deviation (SD). To determine the significance of differences between baseline and post-treatment data, parametric data were analyzed using pairwise t-tests, while nonparametric data were analyzed using Wilcoxon signed-rank tests. The correlation between EC_CO_ and LiD_CO_ was examined using least squares regression analysis, the bias was calculated as LiD_CO_ minus EC_CO_, and the normality of this bias was also evaluated. Additionally, the relative bias in percentage was considered to account for the wide range of cardiac output (CO) studied, based on the cardiovascular effects from the interventions [44]. A positive relative bias (%) indicated underestimation by EC_CO_, while a negative bias indicated overestimation. The limits of agreement (LOA) were expressed as the relative bias ± 1.96 × SD with a 95% confidence interval. A relative bias less than 30% was considered acceptable, and the percentage of observations with a relative bias exceeding 30% was reported for EC [44]. For measuring precision and accuracy for EC, the Lin’s concordance correlation (ρ_c_) was used [45]. Bland–Altman (BA) analysis was employed to demonstrate agreement between EC_CO_ and LiD_CO_ values [46,47], adapting the method of non-uniform differences. Polar plot analysis illustrated the trending pattern and agreement between EC and LiD [48,49]. A *p*-value below 0.05 was deemed statistically significant. Statistical software SAS Version 9.4 (SAS Institute Inc., Cary, NC, USA) was utilized for data analysis, and BA plots were developed using Excel, while polar plots were generated using the polar plot 3 analysis add-in (https://andypope.info/charts/polarplot3.html, accessed on 20 April 2023).

## 3. Results

The entire anesthetic event for all dogs was smooth and without complications. In each dog, the instrumentation for LiD and EC was performed successfully for each dog and no adverse events occurred. No missing data were identified throughout the experiment. Normothermia (37.1 ± 0.3 °C) and normocapnia (40 ± 2 mmHg) were observed in all animals throughout the study. For all dogs, there was no dissimilarity in the total anesthetic duration (*p* = 0.93) and the four treatment times, i.e., DOB (*p* = 0.21), ESM (*p* = 0.54), PHE (*p* = 0.22), and ISO (*p* = 0.44). The timing of the readings between baseline and achievement of hemodynamic goals did not have a significant difference (*p* > 0.05) for all dogs. During DOB, dobutamine dosed at 8.2 ± 1.4 μg/kg/min led to a 48.6 ± 3.8% significant increase (*p* < 0.001) in the LiD_CO_ readings. During ESM, 185 ± 11 μg/kg/min significantly lowered (*p* < 0.001) the LiD_CO_ values by 45.9 ± 8.1%. During PHE, a phenylephrine dosage of 0.4 ± 0.1 μg/kg/min was given to reach MAP of 145 ± 10 mmHg, while ET_ISO_ 3.8 ± 0.3% lowered the MAP values to 39 ± 5 mmHg during the ISO treatment. The percent variation for the LiD_CO_ values between the DOB_baseline_, ESM_baseline_, PHE_baseline_, and ISO_baseline_ readings was 9.6 ± 1.5%. No difference was observed in the blood hemoglobin, packed cell volume, and plasma sodium concentration across treatments during change in hemodynamics, baselines, and also between dogs.

### 3.1. Comparisons between LiD_CO_ and EC_CO_ during the Experiment

For each dog, a CO-paired measurement using the LiD and EC techniques was performed with the four interventions (DOB, ESM, PHEN, and ISO), along with baseline readings before each treatment. This yielded 48 paired observations for six dogs. The LiD_CO_ and EC_CO_ were significantly decreased during the ESM, ISO, and PHEN treatments as compared to the ESM_baseline_ (*p* = 0.029), ISO_baseline_ (*p* = 0.012), and PHE_baseline_ (*p* = 0.033) values, but they were significantly improved for DOB vs. DOB_baseline_ (*p* = 0.025). The mean ± SD relative bias of EC_CO_ in regards to LiD_CO_ was small (−0.67 ± 15.2%), indicating an overall good performance. The LOA for EC_CO_ ranged from −30.4% to 29.1%, with <7% of the observations showing a difference in CO values between two techniques greater than 30%. The percentage error for EC_CO_ was ±31%.

**Figure 4:**
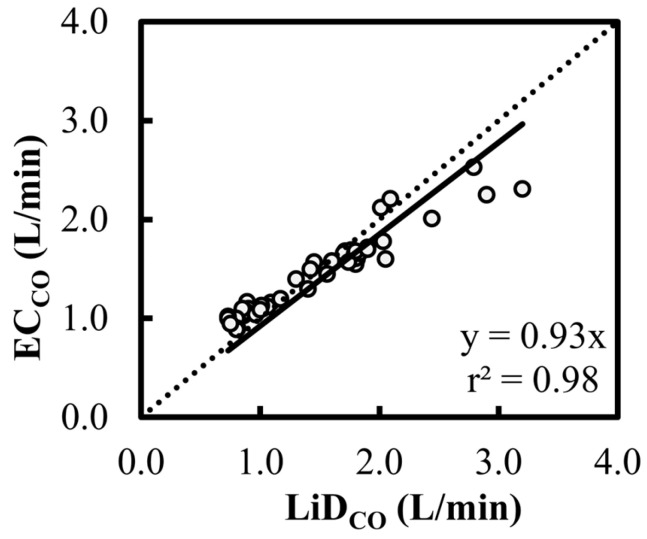
Scatter plot characterizing the cardiac output (CO) estimations using electrical cardiometry (EC_CO_) and lithium dilution (LiD_CO_) in six healthy, anesthetized beagles during various interventions (dobutamine, esmolol, phenylephrine, and high-dose isoflurane) and exhibiting 48 paired observations (circles). The slope of 0.93 and r^2^ of 0.98 for the regression about Y = X (dashed line) suggests a good fit.

A high correlation was observed between the EC and LiD techniques (r^2^ > 0.98) with normally distributed residuals, and the ρ_c_ value of 0.88 indicated very good concordance between the two methods. While the scatter plot for EC_CO_ (Figure 4) suggested that there was no consistent bias for lower values of CO, a consistent underestimation was observed at higher values of CO compared with LiD_CO_, which resulted in a slope about Y = X equal to 0.93. Overall, these findings suggest that EC exhibits promising potential to accurately estimate CO.

**Figure 5:**
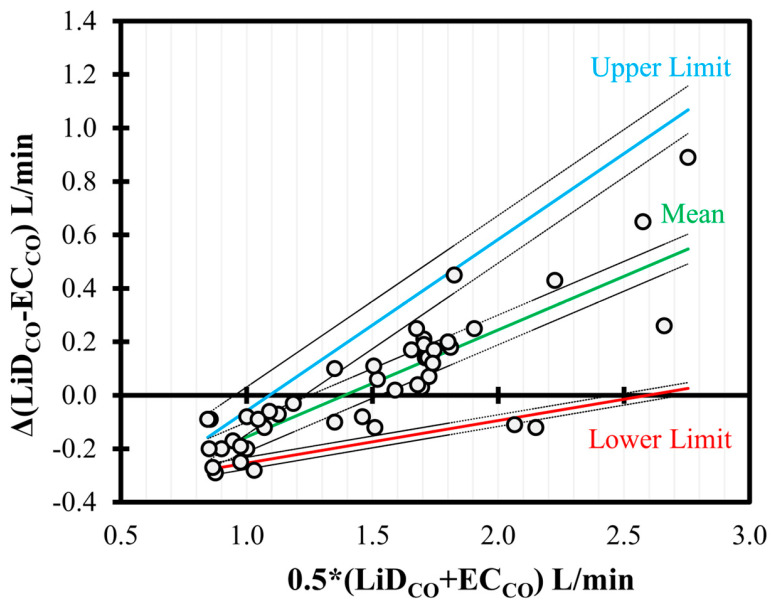
Bland–Altman analysis of non-uniform differences in cardiac output (CO) measurements using electrical cardiometry (EC_CO_) as compared to lithium dilution (LiD_CO_) in six healthy, anesthetized beagles during various interventions (dobutamine, esmolol, phenylephrine, and high-dose isoflurane) and exhibiting 48 paired observations (circles). The individual difference from the mean is denoted by circles, and the non-uniform mean bias is denoted by central line. The solid (mean = green; upper = blue; and lower = red) and the dashed lines represent limits of agreement and the 95% confidence intervals, respectively.

In the BA analysis conducted for EC_CO_ (Figure 5), good agreement was observed but with a significant positive trend (slope = +0.40, intercept = −0.56, *p* < 0.001) between the bias and the mean CO values. Although the proportion of the bias was smaller for low values of CO (<2 L/min), consistent underestimation was observed for higher CO data.

**Figure 6:**
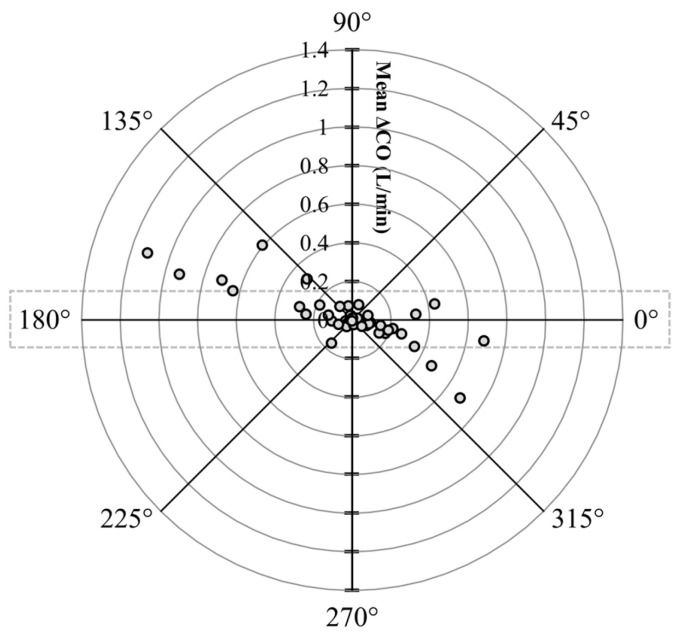
Polar plot characterizing changes in cardiac output (CO) measurements evaluated using electrical cardiometry (EC_CO_) as compared to lithium dilution (LiD_CO_) in six healthy, anesthetized beagle dogs across four treatments (dobutamine, esmolol, phenylephrine, and high-dose isoflurane), thus yielding 48 paired observations. The agreement limits (i.e., 10% = 0.154 L/min as mean LiD_CO_ = 1.54 L/min) are shown through the dotted rectangle. The absolute values of the mean change in CO ([ΔLiD_CO_ + ΔEC_CO_]/2) are represented as the distance from the center, and the disagreement is represented by the angle from the horizontal 0° radial axis. Considering that <20% of the values were outside of the limits of good agreement, a good trend for EC_CO_ was observed.

The polar plot highlighted a good trend pattern for EC_CO_ (Figure 6) across a wide distribution of CO values, as <20% of the values were outside of the limits of good agreement (i.e., 10% mean CO = 0.154 L/min as mean LiD_CO_ = 1.54 L/min).

### 3.2. Impact of Pharmacological Interventions on EC-Derived Variables during the Experiment

The means ± SDs of various hemodynamic parameters during four treatments is shown in Table 1. No change was observed in the HR_EC_, SV_EC_, FTC, ICON™, STR, PEP, and LVET values between the DOB_baseline_, ESM_baseline_, PHE_baseline_, and ISO_baseline_ readings in all dogs. During the DOB treatment, SV_EC_, ICON™, and LVET significantly increased as compared to DOB_baseline_, while EC_SVR_, PEP, and STR were observed to decrease (*p* < 0.001). During the ESM treatment, HR_EC_, SV_EC_, ICON™, and LVET significantly decreased, whereas EC_SVR_, PEP, and STR were higher as compared to ESM_baseline_ (*p* < 0.001). The values for FTC were unchanged in the DOB (*p* = 0.56) and ESM (*p* = 0.91) treatments. Once MAP >120 mmHg was seen during PHE, it was observed that HR_EC_, SV_EC_, and FTC significantly declined, while EC_SVR_, ICON™, PEP, and LVET increased as opposed to the PHE_baseline_ measurements (*p* < 0.001). When high ET_ISO_ was administered, HR_EC_, SV_EC_, EC_SVR_, ICON™, and LVET were significantly lower, whereas PEP, STR, and FTC were increased in comparison with ISO_baseline_ (*p* < 0.001).

## 4. Discussion

As clinicians, we expect newer CO techniques to not only demonstrate safety, but also offer validated, accurate, and reproducible measurements over a wide range of CO values in patients. When method comparison studies on CO monitoring are performed, it is proposed that the standard for the percentage error should be met as a criterion for the acceptability of agreement. From a clinician’s standpoint, imposing arbitrary limits to determine the accuracy and precision of CO measurements can be questioned, as simply focusing on their absolute accuracy may not always be the priority. The percentage error, with respect to pulmonary artery thermodilution of ±45%, signifies a more practical approach to acquiring preciseness in clinical settings [50]. Hence, clinicians may benefit from a CO method that may not display accurate values but may still detect true trends. The current study compared LiD_CO_ and EC_CO_ values ranging from 62.4 to 318.8 mL/kg/min, encompassing a broad range of physiologically occurring CO readings. In the present study, the small relative bias with <7% of the observations showing a difference in CO values between two techniques greater than 30%, ±31% percentage error, and concordance of 0.88 indicated the good, acceptable performance of EC in determining CO as compared to LiD. Consistent underestimation was noted for higher LiD_CO_ values. When comparing the two CO measurement technologies, potential time delays must be accounted for; hence, response times and trending analysis need to be reported. Looking at the polar plots, a good trending behavior was exhibited by EC_._

‘Does the CO monitoring significantly influence patient management, care and outcome?’ is a debatable question and an evolving field. Clinicians use pulmonary artery thermodilution as the de facto ‘gold standard’ CO technique, which is widely explored in research and clinical settings across species. The caveats accompanying this method are its level of invasiveness, measurement errors and variability, cost of instrumentation, and reported complications [51]. The limitations of thermodilution aroused curiosity among researchers to develop minimally invasive CO methods such as LiD that can be implemented in clinical and research settings. Measurement of CO via LiD closely compares with intermittent pulmonary artery thermodilution in dogs [21,24], cats [25], pigs [52], foals [22,23], and horses [20], and LiD is incorporated as a reference method to validate other CO monitoring technologies in various animal studies [41,43,53,54,55,56,57,58]. The advantages of LiD make it conducive for use in animals. It does not require right heart catheterization as with thermodilution, simply requiring the placement of a central venous catheter and a peripheral arterial catheter, both of which are routinely placed in the management of critically ill animals. Interestingly, in dogs, LiD_CO_ measurements using the cephalic vein offer reliable, accurate readings in agreement with the central vein, thus further cutting down on the instrumentation phase [59]. Additionally, advanced LiD monitors combine pulse contour analysis with LiD calibrations for continuous estimation of stroke volume and stroke volume variation. The ‘PulseCO’ algorithm converts the arterial waveform beat by beat to a volume equivalent by making autocorrections for compliance and aortic volume. The root mean square method is independent of the waveform morphology and estimates the effective value (roughly 0.7 × original amplitude) of this volume waveform, computing the ‘nominal stroke volume’. This is scaled to an ‘actual stroke volume’ using a human nomogram acquired from the patient’s age, height, and weight, which corrects for the vascular tree compliance for a specific blood pressure [60]. Caution must be exercised during direct application of this nomogram to veterinary species. This is why we chose to perform the LiD technique as a reference standard by referring to previous canine studies [56,57,58].

On every occasion where the LiD method is involved, blood loss is certain, and hemoglobin concentrations are vital for LiD determination. The total number of LiD_CO_ readings per dog carried out in our study corresponding to the main data points only was 16 (2 readings per time point averaged to 1 = 2 × 8 data points = 16 LiD_CO_ values). Most LiD_CO_ measurements were displayed on the monitor within one minute. During this phase, the LiD peristaltic pump withdraws blood from the arterial line at 4 mL/min to bathe the sensor. This results in approximately 7% blood loss in a 10 kg dog, which sounds noteworthy, but is still categorized as ‘minimal’. However, this can become significant in small-sized patients or sick, anemic animals that require several measurements over multiple timepoints. The recommendation to reduce the blood withdrawn in smaller patients could allow just enough blood to reach the sensor and soak it to help stabilize the sensor prior to initiating the pump. Another technical issue is that the power capacity of the battery can impact the blood flow through the pump, leading to inaccurate LiD_CO_ values. Any clots obstructing the arterial catheter can also slow down the rate of blood flow across the sensor, affecting the CO values, which is why it is mandatory to periodically flush the arterial catheter with heparinized saline [21,51]. The LiD monitor recognizes a high signal-to-noise ratio considering that lithium is foreign to the body’s constitution, which enables low doses of lithium to activate the electrode. Serum lithium concentrations were not assessed in the present study, but the total mean cumulative dose of intravenous lithium was too low (0.073 ± 0.008 mmol/kg) to have a pharmacological effect. Multiple lithium injections that do not undergo rapid clearance from the circulation can potentially increase the background interference, thus resulting in overestimation of CO [51,61]. Even though the background lithium concentrations were not evaluated, we do not anticipate this affecting our study data, as the LiD technique and dosing performed were in accordance with the manufacturer’s guidelines. The manufacturer’s maximum recommended total dose (3 mmol) must be exceeded before toxicity ensues [62]. None of our study dogs showed signs of lithium toxicosis, such as spastic tremors, seizures, gastrointestinal disturbances, cardiovascular instability, hematological alterations, and skin lesions [63]. Additionally, alpha_2_ agonists, ketamine, midazolam, and neuromuscular blockers can produce high bias in the LiD voltage sensor, causing it to drift [51,64]; hence, these drugs were avoided in the present study. Clinical scenarios where LiD may be an imprudent choice include arrhythmias, aortic regurgitation, aortic surgery, intra- or extracardiac shunts, pregnancy, and lithium therapy [51].

Our study showed that the mean ± SD relative bias (LOA) between the two techniques (LiD_CO_–EC_CO_) was −0.67 ± 15.2% (−30.4% to 29.1%), with a percentage error of ±31% and an ρc value of 0.88. Consistent underestimation was observed for higher CO values, similar to the findings in a recent canine study [33] that reported a mean ± SD relative bias (LOA) between thermodilution and EC of 27.7 ± 16.8% (−5.1% to 60.5%), a percentage error of ±49.4%, and an ρc value of 0.65, with overall underestimation by EC, but displaying a good trending ability. It is speculated that in hyperdynamic states (high CO caused by an increase in blood volume), there is an increased blood velocity in the vessels that, combined with a low blood viscosity, may convert laminar flow into turbulent flow, leading to chaotic blood flow. These events may disrupt the orientation of the red blood cells and thus affect the thoracic electric bioimpedance, leading to inaccuracies in higher CO values. This could possibly be why EC underestimated CO during dobutamine-induced positive inotropy. In anesthetized piglets, EC was evaluated against transpulmonary thermodilution concerning CO variations initiated by colloid infusion, epinephrine infusion, and exsanguination [34]. The BA analysis indicated a mean difference (SD) of −0.63 L/min (0.64 L/min), with a percentage error of ±82.8%. The EC_CO_ values underestimated the high and overestimated the low transpulmonary thermodilution readings. It was postulated that the extensive areas of adipose tissue and heavy musculature surrounding the cervical area of the piglets could affect instrumentation and functioning of EC. In anesthetized dogs undergoing open-chest cardiac surgery, the overall bias and precision for cardiac index values using pulmonary artery thermodilution and EC were −0.22 ± 0.52 L/min/m^2^, with a LOA ranging from −1.25 to 0.81 L/min/m^2^. The bias was more significant for low cardiac index values. However, the concordance was 88% with a percentage up to 41.2% and a poor trending behavior for EC. The higher error was assumed to be because of species differences with respect to the aortic arch location, skin thickness and resistance, and width of the thorax [29,34]. Findings from the current study signified an overall better performance, highly reproducible measurements, a good trending nature, and a percentage error accepted within published standards when compared to the above studies.

A potential source for constant discrepancies in EC_CO_ estimations could be how the stroke volume is derived using this technology. The Electrical Velocimetry™ model utilizes the ‘volume of electrically participating tissue’ (i.e., V_EPT_) via anthropometric variables (i.e., weight, height) to estimate the volume of electrically participating tissue present in the thoracic area. Patient body weight is a crucial element, and mass-based volumetric equivalents of the thoracic blood volume were derived in healthy and hemodynamically unstable human patients. Hence, direct translation of this patient constant to other species could contribute to inaccurate absolute measurements, variability, and errors being reported in animal studies. This algorithm in EC challenges the traditional thoracic impedance cardiography mechanism by estimating variations in the thoracic electrical bioimpedance as the ohmic equivalent of the mean aortic acceleration [34,35,36]. The main foci are the impedance and conductivity of the erythrocytes in the aortic blood during systole and diastole. Less important components, such as lung tissue, thoracic fluid, pulmonary and venous blood, gas, and surrounding tissues, are eliminated from the calculations, unlike in thoracic impedance cardiography, which altogether improves the performance of EC [35,36]. The advantages of EC include the following: (i) it can be used in awake small animals due to its noninvasiveness; (ii) it is user-friendly and easy to train; (iii) there is no need for repetitive calibrations; (iv) simple instrumentation is achievable in <10 min; (v) effortless data acquisition and storage; and (vi) it is portable and trouble-free to carry around. The utilization of EC in clinical veterinary patients may be restricted by the following: (i) the size of the animal as the leads may not be long enough to cover wider distances between the neck and thorax, as seen in large animals; (ii) arrhythmias; (iii) extreme motion and heavy panting; (iv) magnetic resonance imaging units; (v) surgical electrocautery; (vi) excessive noise interference from mechanical ventilation; (vii) ascites leading to misinterpretation of body weight; (viii) upper abdomen surgeries where manipulation can impact the thoracic bioimpedance; and (ix) thoracic surgeries.

Acute changes in contractility, afterload, or both were induced by the pharmacological interventions, and the EC-acquired hemodynamic variables that responded quickly and accurately relayed crucial cardiovascular information. Literature specific to variables such as FTC, ICON™, STR, PEP, and LVET obtained from EC technology is lacking in canines, which is why results from the present study could not be compared to previous studies. However, except ICON™, the other EC-derived variables can be validated using transesophageal echocardiogram. The FTC is a systolic component that is calculated by measuring the systolic flow time along with the correction of heart rate. It is proportional to preload and inotropy but can also share an inverse relationship with the systemic vascular resistance [33,37,65]. The current study findings support these theories. During the dobutamine infusion, it is possible that the beta_1_-adrenergic receptor agonism resulted in improved myocardial contractions and ventricular output. Blockade of this receptor by esmolol led to antagonistic negative inotropy with a simultaneous drop in FTC values. The increase in EC_SVR_ during the PHE treatment and the decrease during the ISO treatment corresponded to coinciding lower and higher FTC values, respectively. The ICON^TM^ index is known to directly capture negative or positive inotropic events [33,35,36], which was also proven in our study, where the ESM and ISO treatments caused low ICON^TM^ values, whereas the DOB treatment amplified this index. Interestingly, we also observed an increase in ICON^TM^ during phenylephrine infusion. We hypothesize that the physiologic basis of this finding may be the ‘Anrep effect’. The sudden increase in afterload during the PHE treatment, as confirmed by the dramatic rise in the EC_SVR_ values, could possibly result in a reduced stroke volume but a higher end-systolic volume with a secondary increase in the end-diastolic volume. Hence, extra blood was left within the ventricle after ejection, which augmented the venous return and ventricular filling. This train of events can cause a tangential increase in preload, boosting sarcomere stretch and the force of contraction [66].

The PEP is the elapsed time between the left ventricular electrical systole and the beginning of ventricular ejection, and is highly influenced by beta_1_-adrenergic receptor-mediated sympathetic activity [33,35,36,67]. Highly determined by the pressure differences across the aortic valve, LVET denotes the time interval between opening and closing of the aortic valve. Both these variables can be influenced by the left ventricular ejection fraction, stroke volume, and heart rate. The STR is a ratio of the electrical and mechanical components during systole equivalent to PEP/LVET [33,35,36,67]. To dilute the HR’s influence on PEP and LVET, the STR values may be analyzed instead, which represent the strength and efficiency of cardiac tissue [67]. Dobutamine activates the cardiac contractile function, permitting the ventricle to produce more pressure at a given left ventricular volume, thus increasing the ejection velocity, ejection fraction, and stroke volume. These events may cause lower PEP and higher LVET values, yielding reduced STR readings. Probably, the opposite is true with esmolol and high-dose isoflurane, which have a negative inotropic effect. Since afterload is the resistance against which the ventricle contracts, the left ventricular pressure must exceed the aortic diastolic pressure so that the aortic valve can open. When phenylephrine increased the afterload, PEP was prolonged as the time taken for the ventricular pressures to rise above the aortic pressure was longer. The baroreceptor reflex-mediated compensatory decrease in heart rate could also affect PEP and LVET during the PHE treatment. Further studies are imperative to decide on the utility of EC-derived variables in clinical canine patients during the use of inotropes and vasopressors to treat hemodynamic instability under general anesthesia.

There were several limitations in our study design. Small sample size prevented us from developing receiver operating characteristic curves, which is why the predictive values, cut-offs, sensitivity, and specificity for the EC-acquired variables could not be analyzed. The low number of timepoints for analysis restricted robust comparisons between LiD and EC. Our main focus was on the cardiovascular status; hence, we created a controlled experimental environment that prevented hypothermia/hyperthermia, hypocapnia/hypercapnia, sympathetic stimulation, and light plane of anesthesia from impacting the study data. Moreover, the study population was healthy animals. We are aware this does not reflect a routine clinical picture; hence, EC requires further investigation in anesthetized canine patients with systemic diseases undergoing a variety of surgeries or procedures.

## 5. Conclusions

In healthy, anesthetized dogs subjected to treatments affecting the hemodynamics, our study findings support a good agreement between the EC_CO_ and LiD_CO_ measurements, with consistent underestimation by LiD_CO_ during the hyperdynamic phase where the CO values are high. The mean ± SD relative bias of EC_CO_ in regards to LiD_CO_ was small (−0.67 ± 15.2%), indicating a favorable performance. The value of ρc was 0.88, and the percentage error was ± 31%, which was deemed acceptable as per the standards published for CO comparison studies. Fewer than 7% of the observations showed a difference in CO values between two techniques greater than 30%. The polar plot exhibited a good trend pattern for EC_CO_ with <20% of the values lying outside the LOA. On the other hand, the EC-derived variables, i.e., EC_SVR_, FTC, ICON^TM^, PEP, LVET, and STR, followed variations in CO, inotropy, and vasomotor tone occurring due to the interventions administered. These parameters may be of importance in leading therapeutic decisions for selecting inotropes, vasopressors, and fluid therapy during cardiovascular complications, and their clinical assessment will be valuable. Electrical cardiometry seems to be a promising noninvasive method that may provide direction in anticipating, diagnosing, and treating cardiovascular complications perioperatively, thus enhancing patient monitoring and anesthetic management in dogs.

## Figures and Tables

**Table 1:** Mean ± standard deviation of the standard parameters, lithium dilution cardiac output (LiD_CO_) measurements, and electrical cardiometry (EC)-derived indices: heart rate (HR_EC_), stroke volume (SV_EC_), systemic vascular resistance (EC_SVR_), corrected flow time (FTC), contractility index (ICON™), systolic time ratio (STR), pre-ejection period (PEP), and left ventricular ejection time (LVET), recorded in six healthy, anesthetized beagles subjected to four randomized pharmacological interventions (dobutamine, esmolol, phenylephrine, and high-dose isoflurane). Baseline values before all treatments were carried out (DOB_baseline_, ESM_baseline_, PHEN_baseline_, and ISO_baseline_).

Variable	DOB_baseline_	DOB	ESM_baseline_	ESM	PHE_baseline_	PHE	ISO_baseline_	ISO
LiD_CO_ (L/min)	1.95 ± 0.46	2.92 ± 0.24 *	1.79 ± 0.27	0.99 ± 0.32 ^†^	1.90 ± 0.22	0.81 ± 0.16 ^‡^	1.76 ± 0.21	0.71 ± 0.29 ^§^
MAP (mmHg)	78 ± 7	92 ± 5 *	81 ± 6	61 ± 8 ^†^	84 ± 7	145 ± 10 ^‡^	71 ± 6	39 ± 5 ^§^
ET_ISO_ (%)	1.5 ± 0.1	1.5 ± 0.1	1.5 ± 0.1	1.5 ± 0.1	1.5 ± 0.0	1.4 ± 0.0	1.5 ± 0.1	3.8 ± 0.3 ^§^
EC-derived								
HR_EC_ (beats/min)	92 ± 5	99 ± 6	99 ± 6	85 ± 7 ^†^	103 ± 7	72 ± 8 ^‡^	95 ± 8	80 ± 6 ^§^
SV_EC_ (mL)	22.9 ± 3.5	31.1 ± 4.2 *	19.2 ± 2.9	10.3 ± 4.9 ^†^	19.6 ± 2.7	12.5 ± 3.0 ^‡^	20.2 ± 4.4	9.4 ± 3.9 ^§^
EC_SVR_ (dynes/s/cm^5^)	3057 ± 276	2346 ± 408 *	3326 ± 454	3902 ± 221 ^†^	3240 ± 259	12,494 ± 563 ^‡^	3296 ± 413	2466 ± 201 ^§^
FTC (ms)	340 ± 9	371 ± 14	321 ± 11	343 ± 20	350 ± 15	274 ± 11 ^‡^	325 ± 18	379 ± 16 ^§^
ICON^TM^	98 ± 14	126 ± 17 *	103 ± 11	62 ± 13 ^†^	95 ± 10	117 ± 9 ^‡^	100 ± 11	69 ± 12 ^§^
STR	0.42 ± 0.11	0.30 ± 0.10 *	0.41 ± 0.10	0.55 ± 0.18 ^†^	0.43 ± 0.12	0.44 ± 0.15	0.41 ± 0.13	0.56 ± 0.11 ^§^
PEP (ms)	114 ± 11	95 ± 10 *	119 ± 15	136 ± 9 ^†^	122 ± 9	141 ± 10 ^‡^	111 ± 9	130 ± 6 ^§^
LVET (ms)	275 ± 17	314 ± 15 *	284 ± 12	246 ± 14 ^†^	286 ± 10	324 ± 13 ^‡^	270 ± 11	229 ± 9 ^§^

* Significant difference (*p* < 0.05) between DOB_baseline_ and DOB; ^†^ significant difference (*p*< 0.05) between ESM_baseline_ and ESM; ^‡^ significant difference (*p* < 0.05) between PHE_baseline_ and PHE; and ^§^ significant difference (*p* < 0.05) between ISO_baseline_ and ISO.

## Data Availability

Data supporting the central findings of this research study are contained within the article. Other data pertaining to studying animals may be available on request and are subjected to evaluation on a case-by-case basis respecting the Virginia Polytechnic Institute and State University regulations and policies on data handling.
